# Editorial

**Published:** 1895-10

**Authors:** 


### Editorial.

When the law creating a State Board of Medical Examiners was passed in January, the confident hope was entertained that it would constitute an effective measure against the entrance into the State of quacks, and medical incompetents of all grades. That each applicant for a license to practice must pass a satisfactory examination before a regularly constituted board, representing the regular profession, the homeopathic and the eclectic schools, seemed most certainly to promise protection against the further maintenance and a sure remedy against the disease of quackery that had for so long been undermining the reputation of the State from a professional standpoint.

How far these hopes were destined to fruition, and in how much the law has been regarded and proven potent, and possessing the deterrent influence so sanguinely looked for, will be seen in the unabated tide of medical pariahs that has steadily poured into this city since the passage of the law. It cannot be in the bounds of reason to suppose that this statute was enacted merely to meet a concerted demand on the part of those engaged in the practice of medicine in the State, irrespective of school, and was never intended to be enforced. Yet, by accident, design or neglect, this seems actually to have been the case.

So far as can be ascertained, there has not one indictment been brought against any person or persons for the illegal practice of medicine, since the law went into "effect." If the authorities of the law, whose plain duty it is to bring these offenders to trial, neglect and ignore this duty, it is the behoof of every man who has regard for the honorable profession of medicine and the high name of the State, to search out these malefactors and drag them, red-handed, before the bar of justice, to demonstrate forever, that though she "moves with leaden feet, she strikes with an iron hand."

Yet all, and possibly, could it be known, not even the moiety of blame is to be laid to supineness on the part of the municipal authorities, or to failure on the part of two of the boards, at least, properly to examine and pass or reject candidates for license. It is more than probable that the fault lies in the existence of three boards, with equal rights and privileges, but with widely differing conceptions of professional ethics and qualifications.

With the regular and homeopathic boards, we have no quarrel, but with that so-called cult of exclusives that would put a premium upon commercialism by electing to a chair in its local institution one who uses the advertising columns of the daily press as his history book, and has as a member of its board, one who would be expelled from any regular medical body on earth for flagrant violation of the code of ethics in advertising. If such be the policy of their leaders, what can be expected of their followers?

The watchmen guard the gates, but unseen of them, there is upon the wall a Rahab with the scarlet cord that makes security an idle boast. This is a most serious menace.

Shall the law be permitted to be emasculated of every element of potency by slothfulness on the part of those who tried so long and faithfully to effect its enactment, and the listless indifference of the profession at large throughout the State?

If prompt remedial measures are not taken by the organized medical bodies of the State and city, the evils, the least of which will be an entire lack of discrimination by the public between a regular ethical practitioner and a quack, will become too firmly fixed for removal.

